# Induction of Central Host Signaling Kinases during Pneumococcal Infection of Human THP-1 Cells

**DOI:** 10.3389/fcimb.2016.00048

**Published:** 2016-04-26

**Authors:** Thomas P. Kohler, Annemarie Scholz, Delia Kiachludis, Sven Hammerschmidt

**Affiliations:** Department Genetics of Microorganisms, Interfaculty Institute for Genetics and Functional Genomics, Ernst-Moritz-Arndt Universität GreifswaldGreifswald, Germany

**Keywords:** *Streptococcus pneumoniae*, phagocytosis, THP-1 cells, cell signaling, p38, Akt, PI3K, ERK1/2

## Abstract

*Streptococcus pneumoniae* is a widespread colonizer of the mucosal epithelia of the upper respiratory tract of human. However, pneumococci are also responsible for numerous local as well as severe systemic infections, especially in children under the age of five and the elderly. Under certain conditions, pneumococci are able to conquer the epithelial barrier, which can lead to a dissemination of the bacteria into underlying tissues and the bloodstream. Here, specialized macrophages represent an essential part of the innate immune system against bacterial intruders. Recognition of the bacteria through different receptors on the surface of macrophages leads thereby to an uptake and elimination of bacteria. Accompanied cytokine release triggers the migration of leukocytes from peripheral blood to the site of infection, where monocytes differentiate into mature macrophages. The rearrangement of the actin cytoskeleton during phagocytosis, resulting in the engulfment of bacteria, is thereby tightly regulated by receptor-mediated phosphorylation cascades of different protein kinases. The molecular cellular processes including the modulation of central protein kinases are only partially solved. In this study, the human monocytic THP-1 cell line was used as a model system to examine the activation of Fcγ and complement receptor-independent signal cascades during infection with *S. pneumoniae*. Pneumococci cultured either in chemically defined or complex medium showed no significant differences in pneumococcal phagocytosis by phorbol 12-myristate 13-acetate (PMA) differentiated THP-1 cells. Double immuno-fluorescence microscopy and antibiotic protection assays demonstrated a time-dependent uptake and killing of *S. pneumoniae* 35A inside of macrophages. Infections of THP-1 cells in the presence of specific pharmacological inhibitors revealed a crucial role of actin polymerization and importance of the phosphoinositide 3-kinase (PI3K) and Protein kinase B (Akt) as well during bacterial uptake. The participation of essential host cell signaling kinases in pneumococcal phagocytosis was deciphered for the kinase Akt, ERK1/2, and p38 and phosphoimmunoblots showed an increased phosphorylation and thus activation upon infection with pneumococci. Taken together, this study deciphers host cell kinases in innate immune cells that are induced upon infection with pneumococci and interfere with bacterial clearance after phagocytosis.

## Introduction

*Streptococcus pneumoniae* is a common colonizer of the upper respiratory tract of human, with increased colonization rates in children and the elderly (Garenne et al., 1992; Bogaert et al., 2004b; Hussain et al., 2005). Beside its role as a harmless colonizer, pneumococci are also a common cause of otitis media, pneumonia, meningitis and sepsis, especially in children under the age of 5 years (Bogaert et al., 2004a; Sleeman et al., 2006). *S. pneumoniae* possesses a wide variety of virulence factors to colonize the host, invade into tissues and to evade the human immune system (Jonsson et al., 1985; Gamez and Hammerschmidt, 2012; Voss et al., 2012). The epithelia of the upper respiratory tract of human represent thereby a physical barrier which needs to be conquered in the process of invasive pneumococcal diseases. Pneumococci therefore release amongst others pneumolysin, neuraminidase, and hyaluronidase to the environment, leading to disruption of connective tissues and extracellular matrices, promoting dissemination of the bacteria into underlying tissues, and the blood system (Kelly and Jedrzejas, 2000; Feldman et al., 2007; Trappetti et al., 2009; Mitchell and Mitchell, 2010). In this scenario, phagocytic cells play an essential role in the recognition and clearance of bacterial infections (van Furth and Cohn, 1968; van Furth et al., 1972). Macrophages represent an important link between the innate and the acquired immune system due to the possibility to phagocytose and digest bacteria and to present part fragments of processed bacteria in association with major histocompatibility complex (MHC) class I or II to T-cells (Greenberg and Grinstein, 2002). Bacterial recognition and uptake by macrophages can be initiated by the activation of different surface receptors. Fc receptor-mediated phagocytosis is initiated by recognition of immunoglobulin G (IgG) opsonized microorganisms. Here, members of the Fcγ receptor family are able to recognize and bind the constant Fc region of IgG molecules that opsonize pathogenic microorganisms (Gomez et al., 1994; Ravetch, 1997). Microorganisms can also be opsonized for complement receptor-mediated phagocytosis by proteins of the complement system, like C3b or C4b, resulting from cleavage of complement factors (Ghiran et al., 2000).

Besides recognition of opsonized microorganisms, cells of the innate immune system have the capability to sense bacteria directly via their target specific molecular structures, the so called pathogen-associated molecular patterns (PAMPs) via pattern recognition receptors (PRRs). These receptors are located on the surface of host cells, intracellularly and are also be secreted (Janeway and Medzhitov, 2002; Iwasaki and Medzhitov, 2015). For the recognition of pneumococci various PRRs have been described, including the C-reactive protein (CRP), members of the toll-like receptor family (TLRs), Nod proteins, the LPS binding protein (LBP), and CD14 (Mold et al., 2002; Weber et al., 2003; Currie et al., 2004; Echchannaoui et al., 2005; Malley et al., 2005). Furthermore, the C-type lectin SIGN-R1 and the scavenger receptor MARCO, both expressed by macrophages, have been described as PRRs important for the recognition of pneumococci (Arredouani et al., 2004; Kang et al., 2004).

However, recognition of bacteria via PAMPs and sensing of opsonized bacteria leads to the initiation of signal transduction cascades catalyzed by different protein kinases ending up in the activation of proteins involved in actin remodeling and therefore phagocytosis (Freeman and Grinstein, 2014). Nevertheless, the molecular process of actin remodeling during phagocytosis is only partially understood and best studied in Fcγ and complement-3 receptor-(CR3) mediated phagocytosis.

The signaling network of protein kinases involved in phagocytosis is rather complex and dependent from the activated surface receptors and cross-talk between different signaling pathways.

Members of the phosphoinositide-3-kinase (PI3K) family are essential for many cellular processes by transducing outside-inside signaling. Amongst others, this signaling leads to the activation of downstream effector pathways, including the reorganization of the cytoskeleton via exchange factors that regulate the small GTPase Rac and activation of the protein kinase C (PKC) as well as the serine/threonine protein kinase B (PKB/Akt) (Hawkins et al., 1995; Katso et al., 2001; Engelman et al., 2006). Moreover, experiments with pharmacological inhibitors of the PI3K (Wortmannin and LY294002) revealed also an essential role in FCγ and complement receptor-mediated phagocytosis (Cox et al., 1999; Aderem, 2003). Blocking of the PI3K leads not to an inhibition of opsonized particle binding or initial actin polymerization but seems to be required for membrane extension and fusion during engulfment (Araki et al., 1996; Cox et al., 1999).

Another important component of intracellular signaling processes is the protein kinase B (Akt), a serine/threonine (Ser/Thr) protein kinase involved in a wide variety of signaling pathways concerning such as cell growth, survival, or cellular metabolism (del Peso et al., 1997; Wullschleger et al., 2006; Manning and Cantley, 2007). Akt is an important downstream target of the PI3K, and represents therefore a mediator of the PI3K activity, as shown for example by blocking Akt activation using the PI3K inhibitor Wortmannin (James et al., 1996). Also, Akt was further shown to be activated during the process of Fcγ receptor-mediated phagocytosis (Ganesan et al., 2004).

A third protein family widely involved in cellular signal transduction pathways is the mitogen-activated protein kinase (MAPK) family. These kinases are also Ser/Thr protein kinases converting extracellular stimuli into a cellular response and they are involved in many physiological processes (Widmann et al., 1999). Examples for conventional MAPKs are the two MAPK isoforms ERK1 and ERK2 which can be activated by a number of different growth factors such as platelet-derived growth factor (PDGF) and epidermal growth factor (EGF) as well as in response to insulin, ligand binding on heteromeric G-protein coupled receptors (GPCR), cytokines, osmotic stress, and microtubule disorganization (Boulton et al., 1990; Raman et al., 2007).

ERK1/2 was shown to be activated during the process of Fcγ-mediated phagocytosis (Karimi and Lennartz, 1998; Fitzer-Attas et al., 2000). Interestingly, ERK1/2 was shown to be inhibited in the early phase of CR3-dependent bacterial phagocytosis as shown by infection of human macrophages with *Francisella tullarensis* (Dai et al., 2013).

The p38 kinases are a further sub-group of the MAPK family, which includes the p38 α, β, γ, and δ kinases. The p38 kinases are highly activated by cytokines and environmental stress and were shown to be critical for the regulation of immune and inflammatory processes (Cuenda and Rousseau, 2007). Bacterial binding to various TLRs initiates bacterial phagocytosis via signaling cascades involving amongst others p38 (Doyle et al., 2004).

A widely-used model cell line for the analysis of macrophage/pathogen interactions are the human monocytic THP-1 cells. The addition of phorbol 12-myristate 13-acetate (PMA) to the growth medium leads to the differentiation of monocytes into macrophage-like phagocytic cells. Due to their availability of Fc receptors, C3b receptors and various pattern recognition receptors, as well as the lack of surface and cytoplasmatic immunoglobulins, THP-1 cells can be used for immunocytochemical studies (Tsuchiya et al., 1980; Matsumoto et al., 1990).

In this study, the human pathogen *S. pneumoniae* and human THP-1cells were used to evaluate the role of the bacterial growth medium on phagocytosis, to study the time-dependent uptake of pneumococci and to visualize the intracellular fate over time within the macrophages. On the other hand Fcγ- and CR-independent signaling mechanisms during phagocytosis of *S. pneumoniae* in differentiated THP-1 cells were analyzed. Therefore, infection experiments in the presence of pharmacological inhibitors of actin polymerization, PI3K and Akt were carried out. Furthermore, cell lysates from different time points of infected THP-1 cells were analyzed by immunoblot analysis to identify the participation of important cellular protein kinases involved in cell signaling during pneumococcal phagocytosis.

## Materials and methods

### Bacterial strains, media, and culture conditions

*Streptococcus pneumoniae* NCTC 10319 (serotype 35A, low encapsulated) (Pracht et al., 2005) was grown in complex medium Todd-Hewitt broth (Oxoid) supplemented with 0.5% yeast extract (THY), defined chemical medium RPMI modified (RPMI_mod_) (Schulz et al., 2014), or on blood agar plates (Oxoid) at 37°C and 5% CO_2_.

### Bacterial growth curves

Bacteria were plated from cryo cultures on blood agar (Columbia agar with sheep blood, Oxoid) and incubated at 37°C and 5% CO_2_. THY medium or RPMI_mod_ (40 ml in polypropylene tubes) was inoculated with freshly grown bacteria to an initial OD_600_ = 0.1–0.15 and incubated without agitation in a water bath at 37°C. Growth was monitored at appropriate time points by measuring absorbance at OD_600_ (BioPhotometer, Eppendorf).

### Phagocytosis experiments

Monocytic THP-1 cells were seeded in 24-well plates (2 × 10^5^ cells per well in RPMI-1640 supplemented with 10% heat inactivated FCS in a volume of 1 ml) and differentiation was stimulated by the addition of 200 nmol/ml phorbol 12-myristate 13-acetate (PMA) (Sigma-Aldrich). Prior to infection THP-1 cells were incubated for 72 h at 37°C and 5% CO_2_. Pneumococci cultured in RPMI_mod_ to mid-log phase (OD_600_ = 0.35–0.45), were centrifuged and washed with infection medium (RPMI-1640, PAA) containing 1% heat inactivated fetal bovine serum (Gibco).

THP-1 cells were infected with *S. pneumoniae* using a multiplicity of infection (MOI) of 50 bacteria per phagocyte at 37°C and 5% CO_2_ in infection medium. Bacteria were slightly centrifuged (2 min, 200 × g) onto the cells to initiate a simultaneous contact with phagocytes. Post infection, phagocytes were washed with infection medium and subsequently incubated with Penicillin G (100 units/ml, Sigma-Aldrich) and Gentamicin (0.1 mg/ml, Sigma-Aldrich) for 1 h at 37°C and 5% CO_2_. After washing, the phagocytes were lysed using a 1% saponin solution. The colony forming units (cfu) of released intracellular pneumococci was determined by plating the bacteria in appropriate dilutions on blood agar plates (Hermans et al., 2006; Noske et al., 2009; Hartel et al., 2011).

Phagocytosis was also analyzed in the presence of the pharmacological inhibitors of PI3-kinase (Wortmannin, 50 nM and LY294002 50 μM, ENZO Life Sciences), Akt kinase (Akt-inhibitor VIII, 2.5 and 5 μM, Calbiochem), and actin polymerization (Cytochalasin D, 0.125 and 0.25 μM, ENZO Life Sciences).

### Double immunofluorescence staining

Pneumococci attached to or phagocytosed by PMA-differentiated THP-1 cells were visualized by double immunofluorescence microscopy (DIF). Therefore, THP-1 cells (2 × 10^5^) were seeded on sterile glass cover slips (12 mm, Hartenstein) and cultured at 37°C and 5% CO_2_, 72 h prior to the infection (in the presence of 200 nmol/ml PMA) and infected as described above. Post-infection, THP-1 cells were washed with infection medium to remove unbound bacteria and then fixed with 1% paraformaldehyde (Roth). After blocking with 5% bovine serum albumin (BSA, Roth), extracellular bacteria were stained using a polyclonal anti-pneumococcal serum (1:200) and secondary goat anti-rabbit IgG coupled to Alexa-Fluor-488 (1:500, Abcam). Intracellular pneumococci were stained with Alexa-Fluor-568 goat anti-rabbit IgG (1:500, Abcam) after permeabilization of the THP-1 cells with 0.1% Triton-X-100 (Sigma) (10 min, room temperature) and pneumococcal antiserum as primary antibody (1:200). For the statistical analysis 100 cells per experiment and time point were analyzed for the number of intracellular bacteria.

### SDS-page and immunoblotting

To analyze the phosphorylation status of selected host kinases during pneumococcal infection, cell lysates were prepared as followed. THP-1 cells (1 × 10^6^) cells were seeded in 6-well plates in a volume of 2 ml/well. Cells were infected as described above and infection was stopped at different time-points by washing cells with ice-cold infection medium, following cell disruption by the addition of Triton-X-100 lysis buffer (10 mM TRIS, 100 mM NaCl, 1 mM EDTA, 1 mM EGTA, 1 mM NaF, 20 mM Na_4_P_2_O_7_, 2 mM Na_3_VO_4_, 0.1% SDS, 1% Triton-X-100, 10% glycerin, 0.5% sodium deoxycholate) supplemented with a protease inhibitor (Complete^®^, Roche). Afterwards, plates were incubated on ice for 10 min followed by sonication (2 × 30 s). After centrifugation (18,234 × g, 10 min, 4°C) protein concentration of the supernatant was determined using the Bradford assay (Sigma) and stored at −20°C. Cell lysates (50 μg/sample) were separated by sodium dodecyl sulfate polyacrylamide gel electrophoresis (SDS-PAGE) on 12% gels. Proteins were transferred on a nitrocellulose membrane (GE Healthcare) by semi-dry blotting. After transfer, the membrane was blocked overnight at 4°C in TBS + 5% skim milk (Roth). Immunodetection was carried out using specific primary and horse radish peroxidase-(HRP)-conjugated secondary antibodies (listed in Table 1). A primary antibody against glycerinaldehyde-3-phosphate dehydrogenase (GAPDH) was used as a loading control. Luminol was used as substrate for HRP. Detection was carried out using a chemiluminescence-detecting camera (ChemoCam, INTAS).

**Table 1:** **Antibodies used in this study**.

**Antibody**	**Dilution**	**MW [kDa]**	**Source**	**Company**
Akt Antibody	1:500	60	Rabbit	Cell Signaling (#9272)
Phospho-Akt (Ser473)	1:500	60	Rabbit	Cell Signaling (#9271)
GAPDH polyclonal antibody	1:50,000	36	Goat	Abnova (#PAB6637)
p44/42 MAPK (ERK1/2) (137F5)	1:1000	42, 44	Rabbit	Cell Signaling (#4695)
Phospho-p44/42 MAPK (ERK1/2) (Thr202/Tyr204)	1:1000	42, 44	Rabbit	Cell Signaling (#9101)
P38 MAPK	1:1000	43	Rabbit	Cell Signaling (#9212)
Phospho-p38 MAPK (Thr180/Tyr182)	1:1000	43	Rabbit	Cell Signaling (#4511)
Goat Anti-Rabbit IgG (H+L) (Clone: pAb)-HRPO	1:500–1:2500	None	Goat	Dianova (#111-035-045)
Polyclonal Rabbit Anti-Goat IgG/HRP	1:2000	None	Rabbit	Dako (#P044901-2)
Goat Anti-Rabbit IgG H&L (Alexa Fluor^®^ 488)	1:500	None	Goat	Abcam (#ab150077)
Goat Anti-Rabbit IgG H&L (Alexa Fluor^®^ 568)	1:500	None	Goat	Abcam (#ab175471)

### Statistics and quantitative analysis of immunoblots

All data are reported as the mean ± SD. Statistical analysis was performed with unpaired, two-tailed Student's *t*-test using GraphPad Prism^®;^ software v5.01. In all analyzes a *p* < 0.05 was considered statistically significant.

The image processing program ImageJ was used for quantificational analysis of immunoblots by densitometry (Girish and Vijayalakshmi, 2004; Schneider et al., 2012).

## Results

### Influence of bacterial growth medium on pneumococcal phagocytosis by THP-1 cells

**Figure 1:**
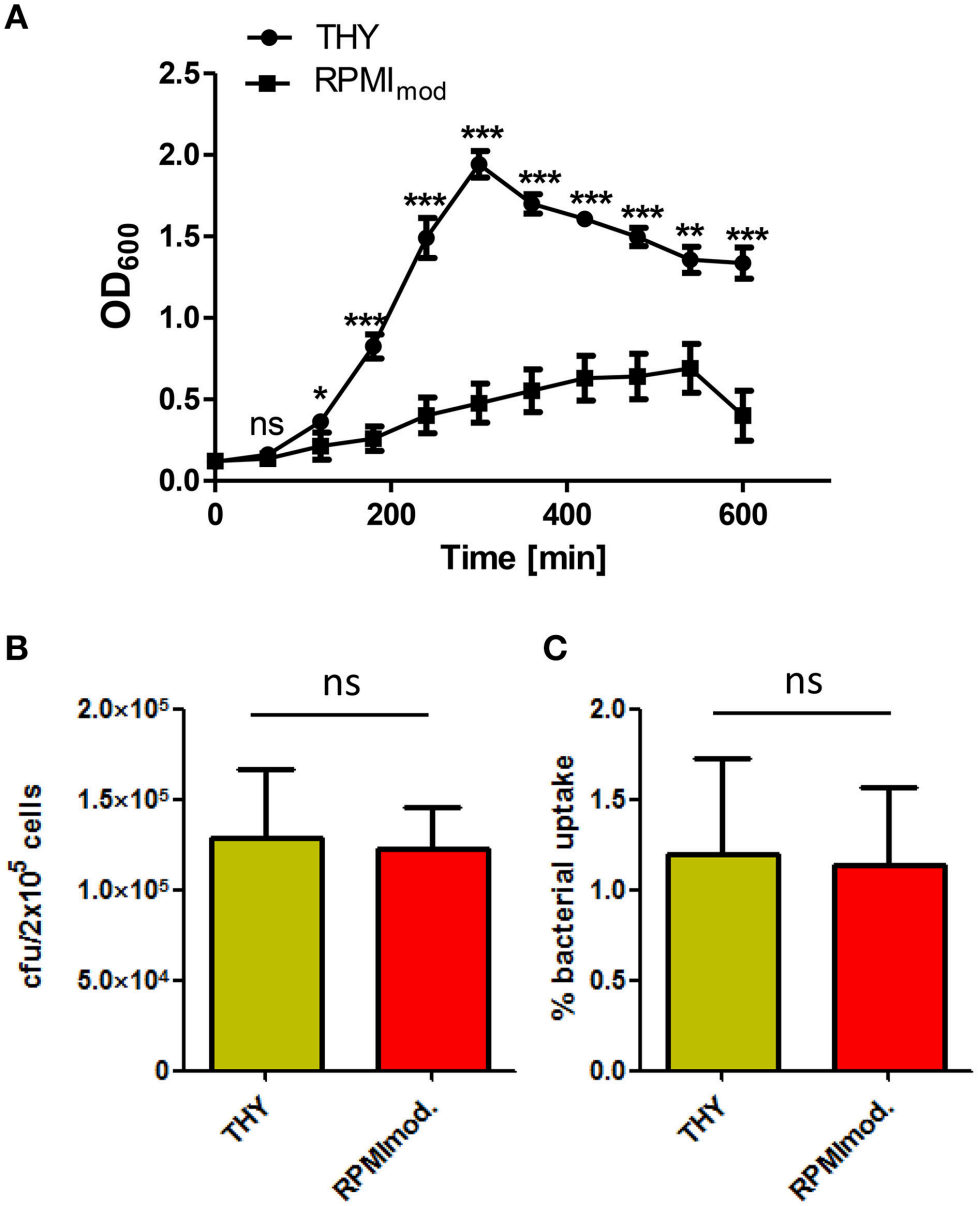
**Influence of the cultivation medium on pneumococcal phagocytosis. (A)** Growth curves of *S. pneumoniae* 35A grown in complex medium (THY) or chemically defined medium (RPMI_mod._). The significance of the THY-medium is based on the difference to RPMI_mod_. **(B,C)** Phagocytosis of *S. pneumoniae* 35A after growth in THY or RPMI_mod_. PMA-differentiated THP-1 cells (2 × 10^5^) were infected (30 min) with a pneumococcal MOI of 50 at 37°C and 5% CO_2_. After infection, extracellular pneumococci were killed by the addition of Gentamycin and Penicillin G (incubation at 37°C and 5% CO_2_ for 1 h). The number of intracellular bacteria was determined by plating cell lysates on blood agar and visualized as recovered cfu **(A)** or % bacterial uptake of the infection dose **(B)**. Values represent means ± SD of at least three independent experiments. ns = not significant, *p* > 0.5; ^*^p < 0.05; ^**^p < 0.01; ^***^p < 0.001.

To assess the impact of the bacterial growth medium on pneumococcal phagocytosis by PMA-differentiated THP-1 cells, *S. pneumoniae* were grown in complex medium (THY) or chemically defined medium (RPMI_mod_)prior to infection. The growth curves of *S. pneumoniae* in the different growth media (Figure 1A) showed a delayed growth in the chemically defined medium in comparison to the complex medium, which is probably due to the high adaptation of pneumococci to the overall nutrient availability in RPMI_mod_. In consequence, pneumococci cultured in THY showed an earlier achievement of the stationary phase and a higher optical density.

Pneumococci used in infection assays were harvested from the exponential growth phase (OD_600_ = 0.35–0.45). PMA-differentiated THP-1 cells were infected for 1 h. The number of recovered intracellular surviving bacteria was determined by the antibiotic protection assay. The results revealed no significant influence of the bacterial growth medium on pneumococcal uptake by PMA-differentiated THP-1 (Figures 1B,C).

### Kinetics of *s. pneumoniae* phagocytosis by THP-1 cells

**Figure 2:**
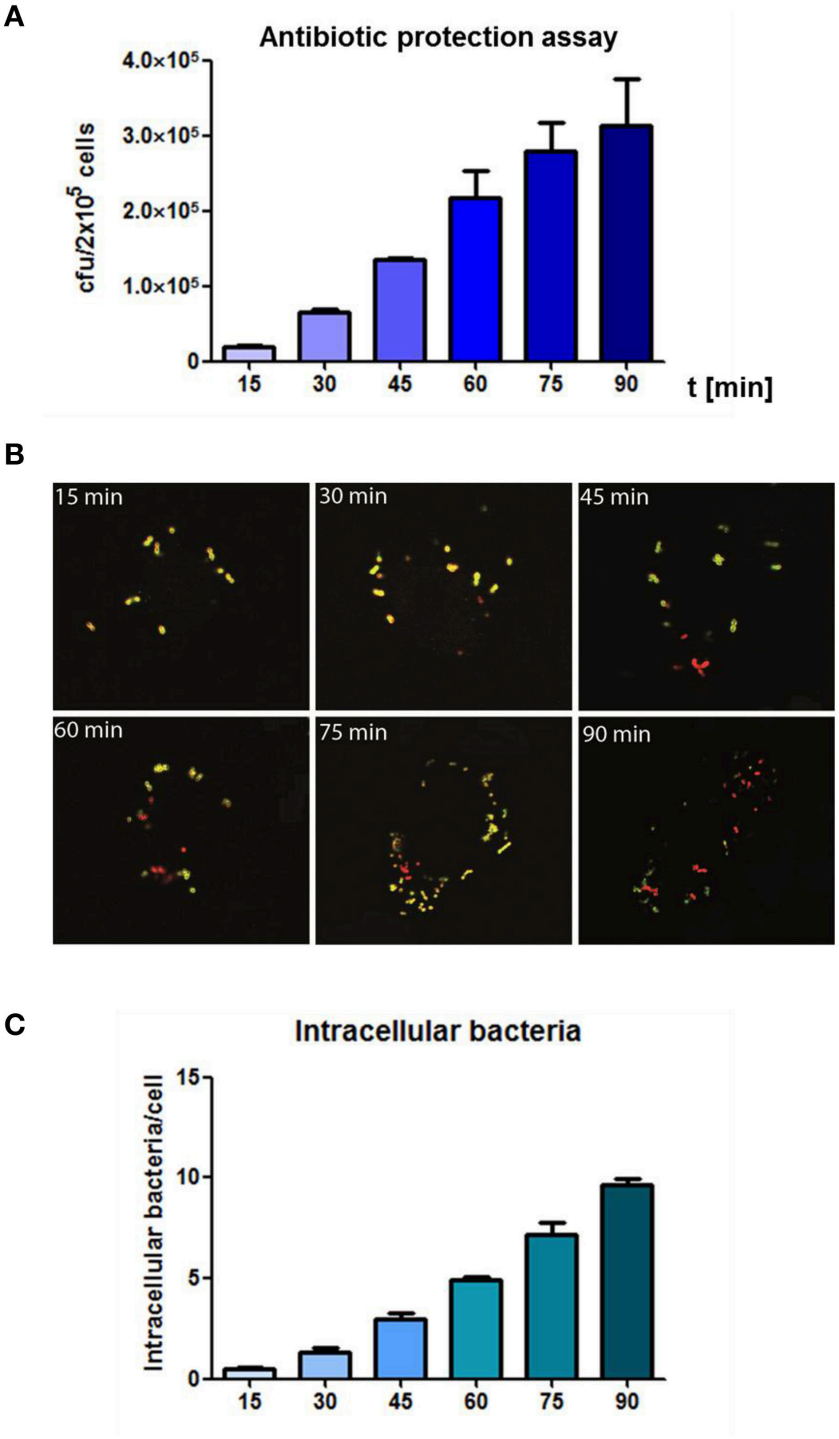
**Analysis of the time-dependent phagocytosis of ***S. pneumoniae***. (A)** Antibiotic protection assay: PMA-differentiated THP-1 cells (2 × 10^5^) were infected with exponential grown *S. pneumoniae* 35A (MOI = 50). The infection was stopped at different time points (15–90 min) by adding of Penicillin G and Gentamicin. Intracellular recovered viable pneumococci were quantified after plating cell lysates on blood agar and enumeration of cfu. **(B)** Double immunofluorescence staining of THP-1 cells at different time-points of infection. Extracellular bacteria were stained using polyclonal anti-pneumococcal antiserum and Alexa-Fluor-488 labeled secondary antibody (green) and after permeabilization of cells with 0.1% Triton-X 100 intracellular bacteria (red) were stained using a polyclonal anti-pneumococcal antiserum and secondary Alexa-568-labeled antibody. **(C)** Intracellular pneumococci of 100 infected THP-1 cells on microscopic slides were counted (double immunofluorescence microscopy) at different time points of infection. Values represent means ± SD of at least three independent experiments.

In the results of the antibiotic protection assay a time-dependent increase of phagocytized pneumococci could be observed over time without saturation 90 min post infection (Figure 2A). In the complementary infection assay, adherent extracellular, and ingested intracellular pneumococci were visualized and illustrated using DIF-staining. Similar to the antibiotic protection assay, an increase of intracellular bacteria was monitored over time as shown by representative microscopic images (Figure 2B). The enumeration of intracellular bacteria in 100 infected THP-1 cells using immunofluorescence microscopy confirmed the results of the antibiotic protection assay. This approach also demonstrated a time-dependent increase of intracellular bacteria upon increasing infection times, without reaching saturation 90 min post-infection (Figure 2C).

### Intracellular fate of phagocytized pneumococci

**Figure 3:**
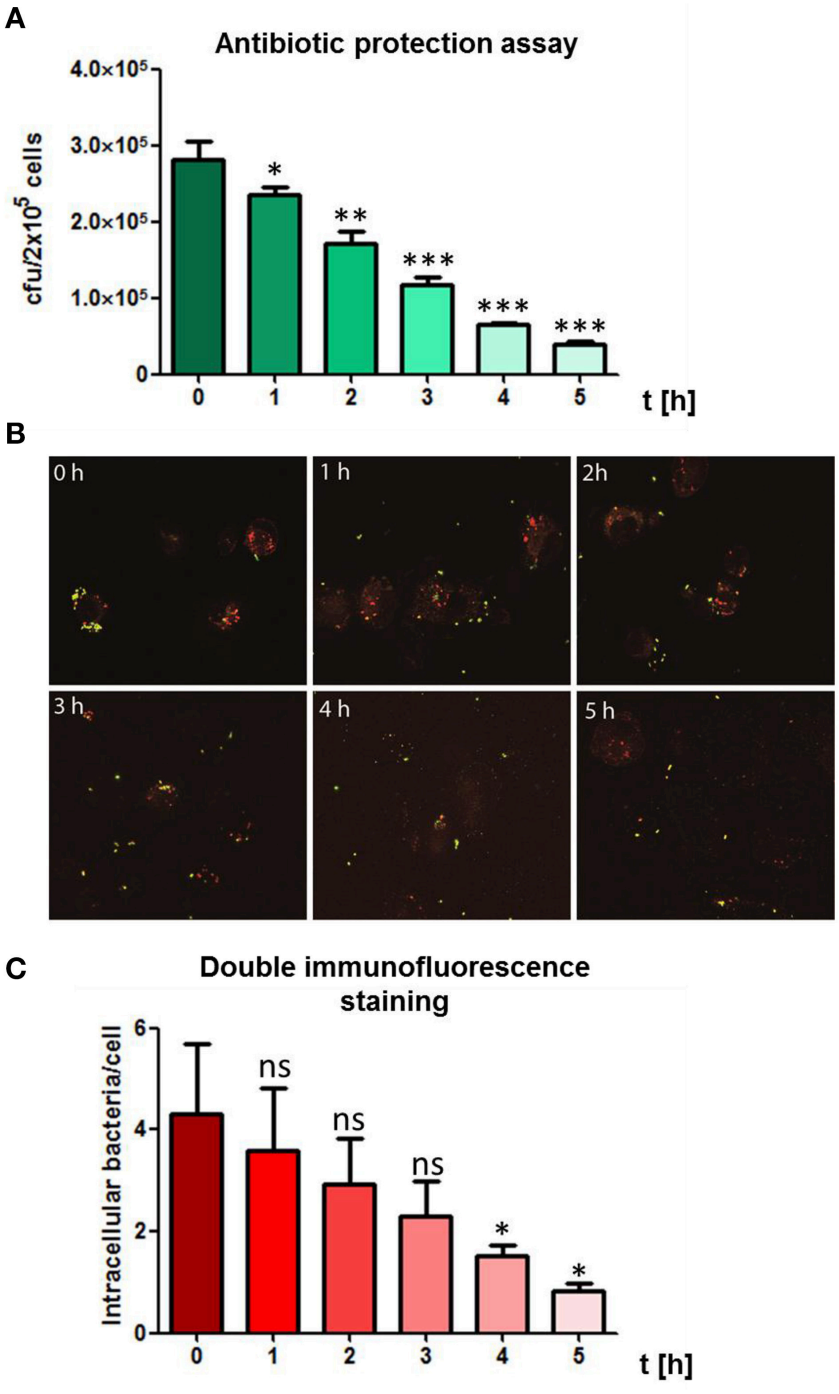
**Time-dependent killing of intracellular pneumococci. (A)** To visualize the bacterial fate of intracellular bacteria, PMA-differentiated THP-1 cells (2 × 10^5^) were infected with exponential grown *S. pneumoniae* 35A (MOI = 50) for 60 min at 37°C and 5% CO_2_. Post infection, extracellular pneumococci were killed by the addition of Gentamycin and Penicillin and subsequently cells were incubated in infection medium. Living intracellular bacteria were quantified by plating cell lysates at different time points post-infection on blood agar. **(B)** Double immunofluorescence staining of infected THP-1 cells at different time-points post infection. Extracellular pneumococci appear in green and intracellular in red. **(C)** Intracellular pneumococci of 100 infected THP-1 cells on microscopic slides were counted after DIF-staining at different time points post-infection. Values represent means ± SD of at least three independent experiments. ns = not significant, *p* > 0.5; ^*^p < 0.05; ^**^p < 0.01; ^***^p < 0.001.

The intracellular fate of *S. pneumoniae* after phagocytosis was analyzed in a time-dependent manner post-infection of THP-1 cells. Extracellular bacteria were killed after infection by the addition of antibiotics. The intracellular number of recovered, viable bacteria was determined after lysis of the THP-1 cells and plating of the bacteria on blood agar plates. The THP-1 cells showed a continuous killing of the engulfed pneumococci during the evaluation period as determined by a constant decrease of bacterial cfu over time (Figure 3A). Similar, the DIF-staining and fluorescence microscopy confirmed the results of the antibiotic protection assay (Figures 3B,C). Representative images and numbers of intracellular bacteria within infected THP-1 cells confirmed the decrease of intracellular pneumococci over the observed time period. Taken together, these results revealed an effective time-dependent intracellular killing of phagocytized pneumococci by THP-1 cells.

### Inhibition of pneumococcal phagocytosis by inhibitors of central host cell kinases

**Figure 4:**
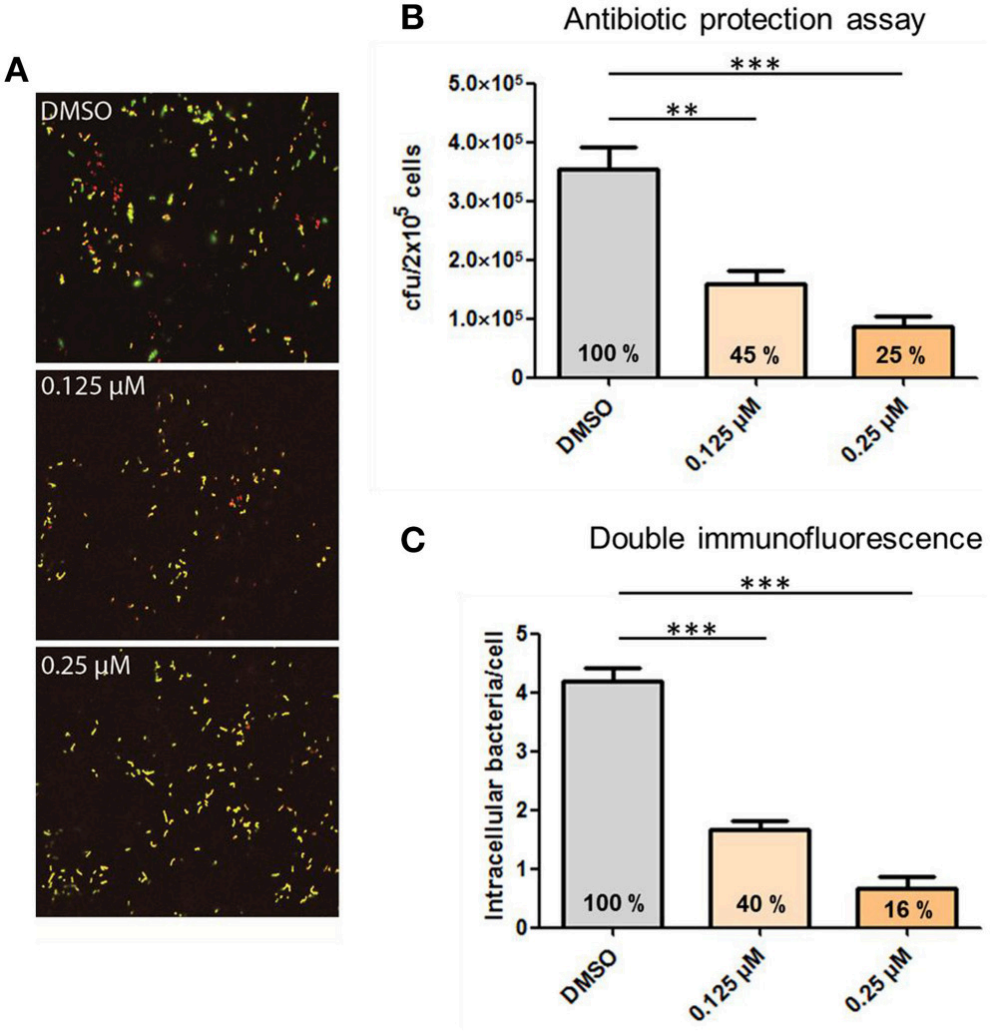
**Influence of the actin polymerization on pneumococcal phagocytosis**. PMA-differentiated THP-1 cells (2 × 10^5^) were infected for 1 h with exponentially grown *S. pneumoniae* 35A (MOI = 50) in the presence of the actin polymerization inhibitor Cytochalasin D (0.125, 0.25 μM). **(A)** Double immunofluorescence staining of THP-1 cells. Extracellular pneumococci appear in green and intracellular in red. **(B)** The number of intracellular recovered and surviving pneumococci cfu was determined using the antibiotic protection assay. **(C)** Intracellular pneumococci of 100 infected THP-1 cells on microscopic slides were enumerated after DIF-staining. Values represent means ± SD of at least three independent experiments. ^**^p < 0.01; ^***^p < 0.001.

Phagocytosis and hence, the rearrangement of the host cell cytoskeleton requires most likely bacterial binding to cell surface receptors, followed by cell signaling through different protein kinases and finally activation of proteins involved in the reorganization of the cytoskeleton. To demonstrate the influence of actin polymerization during phagocytosis, infection experiments were carried out in the presence of Cytochalasin D (CytoD), a potent inhibitor of actin polymerization. PMA-differentiated THP-1 cells were infected with *S. pneumoniae* 35A in the presence of different concentrations of CytoD. DIF- staining and immunofluorescence microscopy were applied to visualize the effect of CytoD on the phagocytosis rate. The addition of increasing concentrations of CytoD resulted in a dose-dependent decline of intracellular pneumococci within the infected THP-1 cells (Figure 4A). To quantify ingested bacteria the number of intracellular bacteria was enumerated in infected cells by DIF-staining and subsequent immunofluorescence microscopy (Figure 4C). A significant, dose-dependent effect of CytoD on the phagocytosis rate (reduction of intracellular bacteria to 40 and 16%) was visible. These results were confirmed with an antibiotic protection assay where the amount of living extracellular bacteria significantly declined dose-dependently (45 and 25%) with increasing concentrations of CytoD (Figure 4B).

**Figure 5:**
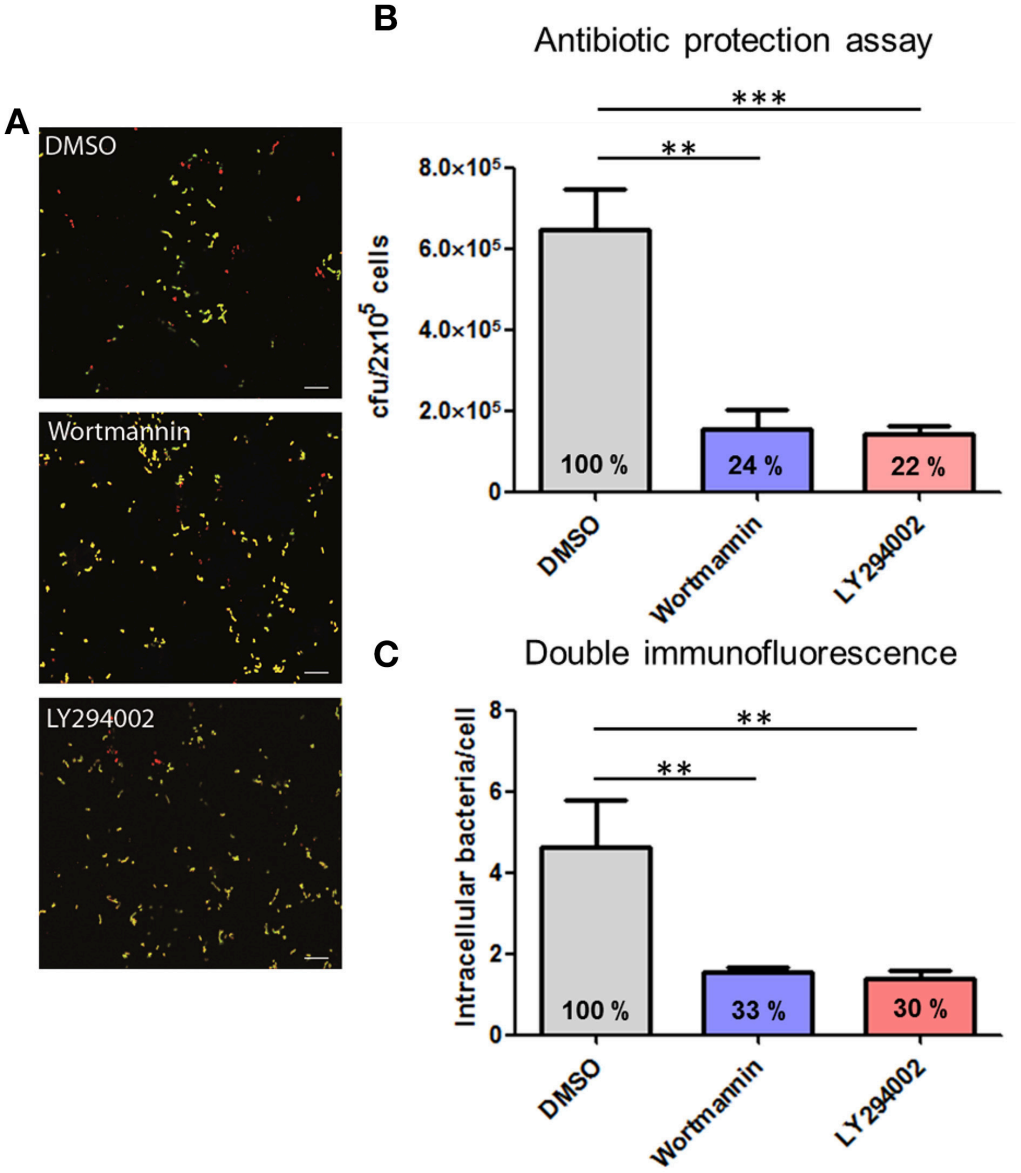
**Influence of the pharmacological inhibitors Wortmannin and LY294002 of the PI3K on pneumococcal phagocytosis**. PMA-differentiated THP-1 cells (2 × 10^5^) were infected for 1 h with exponential grown *S. pneumoniae* 35A in the presence or absence of the PI3K inhibitor Wortmannin (50 nM) or LY294002 (50 μM). **(A)** Double immunofluorescence staining of THP-1 cells. Extracellular pneumococci appear in green and intracellular in red. **(B)** Intracellular cfu was determined by the antibiotic protection assay and intracellular cfu was determined by plating cell lysates on blood agar. **(C)** Intracellular pneumococci of 100 infected THP-1 cells on microscopic slides were counted after DIF-staining. Values represent means ± SD of at least three independent experiments. ^**^p < 0.01; ^***^p < 0.001.

**Figure 6:**
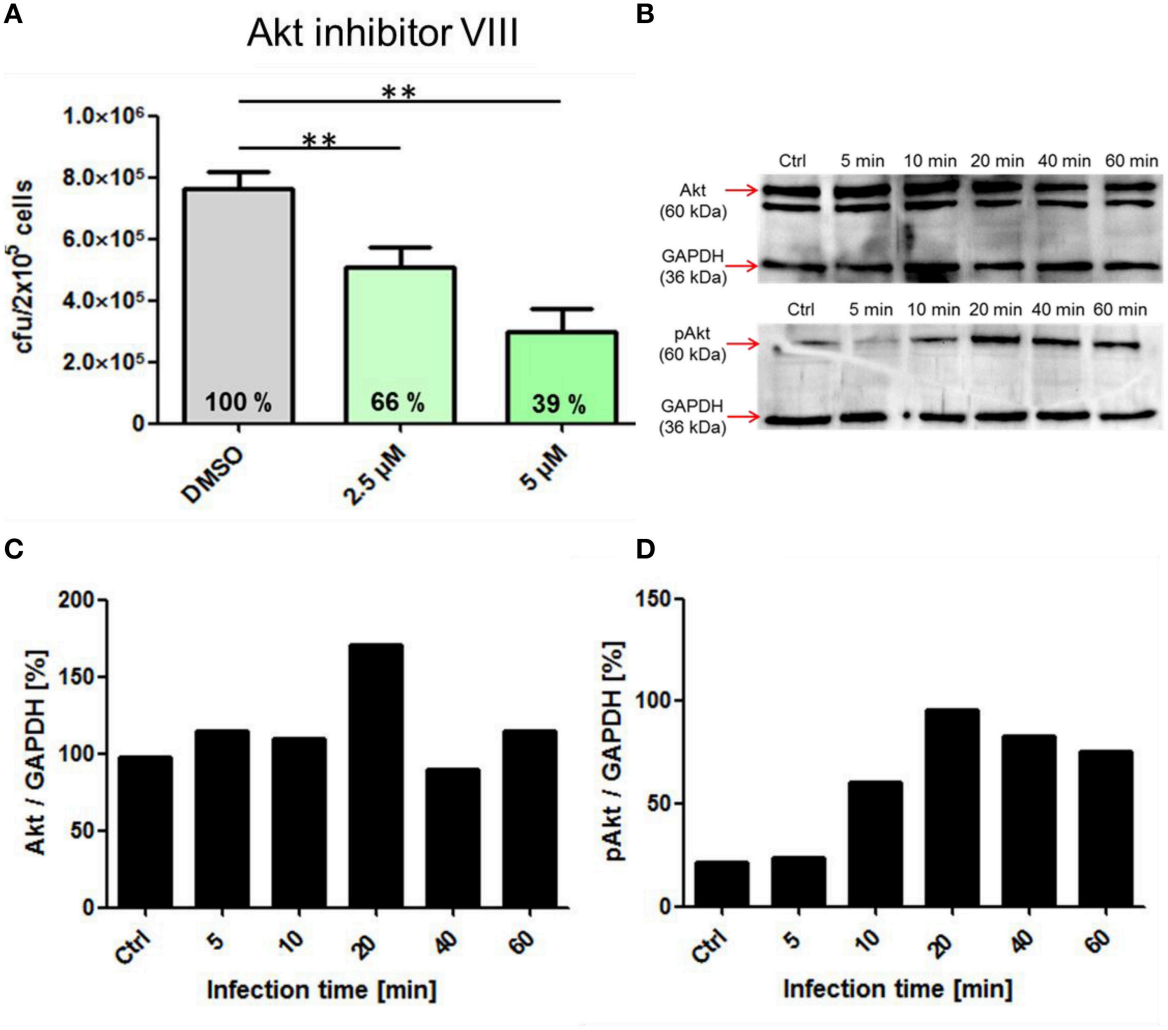
**Influence of the pharmacological Inhibitor Akt inhibitor VIII on pneumococcal phagocytosis and cell signaling. (A)** Intracellular cfu was determined after infection of PMA-differentiated THP-cells (2 × 10^5^) with *S. pneumoniae* 35A in the presence of Akt inhibitor VIII (2.5, 5 μM or DMSO as control) using the antibiotic protection assay and subsequent plating of bacteria on blood agar. **(B)** Immunoblot analysis of total Akt and pAkt. Cell lysates of different time points post-infection (5 to 60 min, Ctrl = uninfected cells) separated using SDS-PAGE and immunoblot analysis. GAPDH was used as a loading control. **(C,D)** Densitometric quantification of total Akt and pAkt in relation to GAPDH by ImageJ. Values of Figure 4A represent means ± SD of at least three independent experiments. ^**^p < 0.01.

This experimental approach was further chosen to analyze the role of the protein kinases PI3K and Akt in the process of pneumococcal phagocytosis by THP-1 cells. The presence of the PI3K inhibitors Wortmannin and LY294002 resulted in a significant inhibition of pneumococcal uptake after 1 h of infection. The microscopic images of the DIF-stained infections (Figure 5A) and counted bacteria within the THP-1 cells (Figure 5C) showed a significant decrease of intracellular bacteria (33 and 30%) compared to the control cells treated only with DMSO. These results were comparable to the results obtained in the complementary antibiotic protection assay (Figure 5B). To analyze the role of the protein kinase Akt, different concentrations of the Akt inhibitor VIII were used during pneumococcal infection of THP-1 cells. The results of the antibiotic protection assay (Figure 6A) in the presence of Akt inhibitor VIII revealed a dose-dependent decrease of viable intracellular pneumococci (66 and 39%) with increasing concentrations of the inhibitor. Taken together, these results reflect the essential role of actin polymerization and the involvement of the protein kinases PI3K and Akt in the Fcγ and CR-independent phagocytosis of *S. pneumoniae* through PMA-differentiated THP-1 cells.

### Time-dependent phosphorylation of selected protein kinases during bacterial phagocytosis

**Figure 7:**
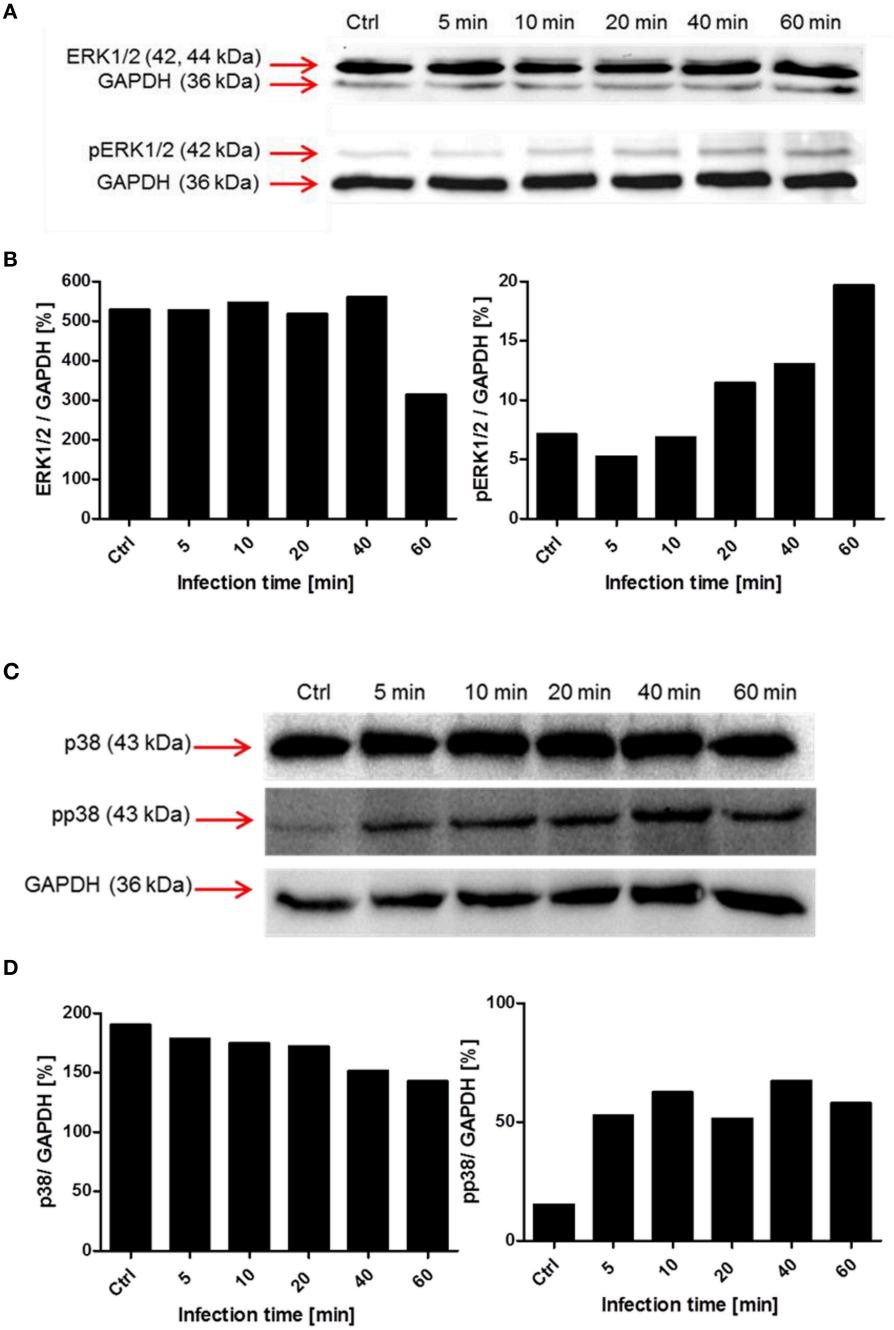
**Time-dependent phosphorylation of mitogen-activated protein kinases during pneumococcal uptake**. Cell lysates of different time points post-infection (5–60 min, Ctrl = uninfected cells) were analyzed using SDS-PAGE and subsequent immunoblot analysis (antibodies used are listed in Table 1). GAPDH was used as a loading control. **(A,B)** Immunoblot analysis and densitometric analysis of ERK1/2 and phosphorylated ERK1/2 (pERK1/2) **(C,D)** Immunoblot and densitometric analysis of p38 and phosphorylated p38 (pp38).

To evaluate the involvement and time-dependent phosphorylation of various protein kinases during pneumococcal infection, PMA-differentiated THP-1 cells were infected with *S. pneumoniae* for different time periods. Cell lysates were used for SDS-PAGE and immunoblot analysis with specific antibodies against phosphorylated and non-phosphorylated forms of different protein kinases (Table 1). The inhibition of Akt in the presence of Akt inhibitor VIII was already demonstrated to be involved in pneumococcal uptake as shown by an antibiotic protection assay (Figure 6A). The immunoblots of Akt/pAkt indicated an increase in Akt phosphorylation 10 min post infection with the highest amount after 20 min (Figure 6B). A densitometric approach was chosen to quantify the obtained results from the immunoblot. The analysis reflects thereby the observed increase of the phosphorylated form of Akt as shown in Figures 6 C,D. Moreover, the phosphorylation of the MAPKs ERK1/2 and p38 during pneumococcal uptake was analyzed (Figure 7). Here the amount of phosphorylated ERK1/2 (pERK1/2) starts to increase 20 min post-infection, whereas the amount of phosphorylated p38 (pp38) increases already after 5 min post-infection.

## Discussion

The innate immune system comprises an enormous arsenal of defense strategies to recognize and eliminate foreign microbes, colonizing or invading the human body. These include amongst others physical barriers like epithelia and mucus, secreted enzymes, antimicrobial peptides, and phagocytes (Someya et al., 2013; Sperandio et al., 2015). A critical function of the immune system is the detection and elimination of invading microbes in normally sterile compartments of the human body. In such a case, professional phagocytes recognize microbes amongst others via PRRs, triggering intracellular signal cascades leading in the end to engulfment and elimination of foreign intruders (Silva, 2010).

In this study, we investigated the interaction of pneumococci with macrophage-like, PMA-differentiated THP-1 cells in the absence of human antibodies or complement. This approach allowed us to analyze the host cell response independently of Fcγ or CR-mediated phagocytosis pathways.

Pneumococci have to adapt to various environmental conditions, including nutrient availability, during colonization of mucosal surfaces in humans and invasive infections. Changes in the nutrient composition or availability results in an alteration of intracellular metabolites, influencing regulatory networks and as a consequence gene expression and protein production (Orihuela et al., 2004; Tang, 2011; Schulz and Hammerschmidt, 2013). Pneumococcal growth in a chemically defined medium reflects much more the *in vivo* situation, in which certain nutrients and/or carbon sources are limited. As shown in the pneumococcal growth curves (Figure 1A), growth was indeed affected due to the limitation of several nutrients. Interestingly, no difference in bacterial uptake through THP-1 cells could be observed using chemically defined (RPMI_mod_) or complex medium (THY). This indicates that the composition of surface-exposed bacterial structures necessary for the recognition and uptake of *S. pneumonia* by THP-1 cells are not substantially changed or reduced due to the composition of the chosen growth media.

Kinetics of pneumococcal phagocytosis by THP-1 cells were analyzed by DIF-staining and subsequent fluorescence microscopy. With this approach, all intracellular bacteria can be visualized, while no differentiation between living and non-viable bacteria is possible. First intracellular pneumococci within THP-1 cells were detected after 15–30 min of infection. The monitored pneumococcal internalization happened in the same time-frame as carried out with other bacteria like *S. aureus* and THP-1 cells (Miller et al., 2011). A saturation in pneumococcal uptake was not observed within the analyzed time period (15–90 min), suggesting a higher capacity of THP-1 cells to take up bacteria.

A major virulence factor of *S. pneumoniae* is the polysaccharide capsule protecting the bacteria from phagocytosis (Wood et al., 1946). In our experimental approach, the low encapsulated strain 35A was chosen to facilitate recognition of surface exposed PAMPs by macrophage PRRs. Intracellular killing of bacteria in macrophages takes place in the phagolysosome via proteases, antimicrobial peptides and reactive oxygen and nitrogen species (Garin et al., 2001). Whereas, several bacterial species evolved strategies to survive or escape from phagolysosomes, such mechanisms are unknown for pneumococci. However, it was shown that during pneumococcal phagocytosis by dendritic cells, a minor proportion of the pneumococci escape from the intracellular vacuoles and resides in the cytosol (Noske et al., 2009). The reason or mechanisms for the pneumococcal escape from phagosomes is unknown. For experiments regarding the intracellular fate of the phagocytized pneumococci during infection, one time point was chosen. Pneumococci phagocytized after 1 h of infection were nearly completely killed within 5 h post-infection in a time-dependent manner, demonstrating the inability of *S. pneumoniae* 35A to survive within the macrophages. However, a minor amount of remaining intracellular pneumococcal cfu 5 h post-infection could be explained with the outbreak of some of the pneumococci from macrophage phagosomes into the cytoplasm.

The participation of the actin machinery and central cellular protein kinases involved in intracellular signaling pathways was analyzed by pneumococcal infection assays in the presence of specific pharmacological inhibitors and immunoblot analysis of THP-1 cell lysates from different time points of infection. The engulfment of pneumococci into phagosomes requires the recognition of the bacteria via surface receptors on THP1-cells, activation of signal cascades and in the end remodeling of the cytoskeleton. The inhibition of actin polymerization by Cytochalasin D during phagocytosis leads in the conducted experiments to a dose-dependent reduction of pneumococcal uptake up to 75%. These results underline the essential function of cytoskeleton rearrangement in pneumococcal phagocytosis by PMA-differentiated THP-1 cells.

The PI3K is an essential regulator of phagocytosis as shown for Fcγ and CR-mediated phagocytosis (Araki et al., 1996; Cox et al., 1999). The enzyme catalyzes after activation the phosphorylation of phosphatidylinositol 4,5-bisphosphate (PIP2) to phosphatidylinositol 3,4,5-trisphosphate (PIP3) (Domin and Waterfield, 1997). PIP3 was shown to be a key player in signaling pathways controlling phagocytosis (Gerisch et al., 2009). The activation of the PI3K can, besides others, also occur via PRRs as shown for various TLRs (Arbibe et al., 2000; Monick et al., 2001). Also in our experiments, using non-opsonized pneumococci, inhibition of the PI3K by the pharmacological inhibitors Wortmannin or LY294002 leads to strong decrease in pneumococcal phagocytosis. The protein kinase B (Akt) is one of the major signal transducers, activated by PIP3 of the PI3K (Chan et al., 1999). After 10 min of pneumococcal infection, the amount of phosphorylated Akt started to increase, with a maximum 20 min post-infection (Figure 4B). Inhibition of Akt by Akt inhibitor VIII led to a dose-dependent decrease of phagocytized pneumococci within the THP-1 cells. First intracellular bacteria where observed after 15–30 min after infection, which is in concert with the activation of Akt after 10 min, which in turn leads to activation of proteins involved in cytoskeleton rearrangement and therefore engulfment of the pneumococci.

Besides the activation of the PI3K and Akt, we were interested in the activation of the mitogen activated kinases ERK1/2 and p38. The infection of THP-1 cells with *S. pneumoniae* resulted in an increase of pERK1/2 20 min post-infection, without saturation after 60 min. The downstream substrates of ERK1/2 includes amongst others transcription factors, kinases, and cytoskeletal proteins (Yoon and Seger, 2006) involved in proliferation, differentiation, and activation of macrophages (Rao, 2001). Further investigations are needed here, especially with a focus on the role of ERK1/2 in the induction of cytokines during phagocytosis. The second MAPK we focused on is p38. This kinase was shown to be activated by several external stimuli like TNF-α, heat, osmotic shock, or growth factors (Freshney et al., 1994; Rouse et al., 1994). Doyle et al. proposed a model in which the activation of TLRs in macrophages leads amongst others to the activation of p38 resulting in the upregulation of scavenger receptors like MARCO and therefore enhanced phagocytosis (Doyle et al., 2004). In our experiments, the phosphorylation of p38 started within the first 5 min post-infection indicating an important role in the early phase of pneumococcal phagocytosis.

The activation of the aforementioned kinases during pneumococcal infection seems not to be restricted to professional phagocytes. Pneumococci were shown to interact with the cellular polymeric immunoglobulin receptor (pIgR) of respiratory epithelial cells via the pneumococcal surface protein C (PspC). The modulated signaling cascades resulting in uptake of the bacteria involves amongst others the PI3K, Akt, and the MAPKs ERK1/2 (Agarwal and Hammerschmidt, 2009; Agarwal et al., 2010). Furthermore, pneumococci were shown to be taken up by human epithelial pharyngeal cells (Detroit 562) exploiting vitronectin as a bridging molecule to interact with α_v_β_3_ integrins. In addition to the integrin linked kinase (ILK) the PI3K plays an essential role in pneumococcal endocytosis via the vitronectin mechanism (Bergmann et al., 2009).

The analysis of further signaling cascades like the JNK, Src, and focal adhesion kinases is necessary to gain deeper insights into signaling events triggered by pneumococci during phagocytosis.

Moreover, pneumococcal infection of macrophages was shown to contribute to apoptosis (Ulett and Adderson, 2006). Therefore, it could be of great interest to analyze the participation of pneumococcal triggered cell signaling pathways on the induction of apoptosis.

Taken together, we used the model of PMA-differentiated THP-1 cells to characterize the interaction of *S. pneumoniae* with professional phagocytes. The relevance of the bacterial growth medium on phagocytosis as well as the time-dependent uptake and killing was demonstrated. Furthermore, insights into cell signaling processes during bacterial uptake were deciphered by using pharmacological inhibitors and performing immunoblot analysis.

## Author contributions

TK was writing the paper and performed experiments. TK was the supervisor of AS. AS executed experiments, DK executed experiments, SH was writing the paper, supervisor of DK, project leader.

### Conflict of Interest Statement

The authors declare that the research was conducted in the absence of any commercial or financial relationships that could be construed as a potential conflict of interest.
